# LC-MS-Based Untargeted Metabolomics Reveals the Effects of Pyrethrins-Mediated Silver Nanoparticles on the Metabolism of *Solenopsis invicta*

**DOI:** 10.3390/ijms27135821

**Published:** 2026-06-27

**Authors:** Huaxin Cai, Wenzhe Li, Dongxu Wang, Canxia Wu, Jingyang Ni, Yinghua Tong

**Affiliations:** Forestry College, Fujian Agriculture and Forestry University, Fuzhou 350002, China; 15080598856@163.com (H.C.); liwz20000423@163.com (W.L.); 13526518529@139.com (D.W.); wucanxia0125@163.com (C.W.); nijingyangnjy@163.com (J.N.)

**Keywords:** red imported fire ant, pyrethrins, silver nanoparticles, toxicity, metabolism

## Abstract

The red imported fire ant (*Solenopsis invicta* Buren) is a destructive invasive pest, and conventional chemical control faces challenges related to environmental contamination and resistance development, highlighting the need for novel control agents and greener management strategies. In this study, pyrethrins-mediated silver nanoparticles (Pyr-AgNPs) were synthesized via a green route, characterized, and evaluated for their insecticidal activity, environmental stability, and metabolic effects on *S. invicta* workers. Bait bioassays showed that Pyr-AgNPs exhibited high toxicity to *S. invicta*, causing 100% cumulative corrected mortality at 500 mg·kg^−1^ after 9 days of feeding, with a 5-d LC_50_ of 116.83 mg·kg^−1^. Exposure assays further demonstrated that Pyr-AgNPs had good environmental stability and residual efficacy, as bait containing 1000 mg·kg^−1^ Pyr-AgNPs still caused 100% cumulative corrected mortality after 9 days following 96 h of outdoor exposure, significantly outperforming the pyrethrins treatment. LC-MS-based untargeted metabolomic analysis revealed that treatment with Pyr-AgNPs markedly altered the metabolic profile of *S. invicta* workers, with 607 differential metabolites identified, mainly belonging to organic acids and derivatives, lipid and lipid-like molecules, amino acids and peptides, cofactors, and redox-related metabolites. Pathway enrichment analysis indicated that these metabolic disturbances were primarily associated with energy metabolism, redox homeostasis, and membrane lipid metabolism. Overall, these findings provide preliminary mechanistic clues into the toxicity of Pyr-AgNPs and support their potential application in the sustainable management of *S. invicta*.

## 1. Introduction

The red imported fire ant (*Solenopsis invicta* Buren) belongs to Hymenoptera, Formicidae, Myrmicinae, and Solenopsis [1]. This species has been listed by the International Union for Conservation of Nature (IUCN) as one of the “100 Most Invasive Alien Species” [2], causing widespread harm to agricultural production and public health in various countries, while also posing a serious threat to ecological security [3,4,5]. Currently, the control of *S. invicta* primarily relies on traditional chemical insecticides, including pyrethroids, organophosphates, phenylpyrazoles, neonicotinoids, and insect growth regulators used in bait formulations [6]; however, long-term reliance on conventional chemical control can compromise control efficiency through resistance development and other practical limitations [7,8]. Therefore, there is an urgent need to explore novel control agents with high efficacy and improve stability for *S. invicta* management.

In recent years, silver nanoparticles (AgNPs) synthesized via biomolecular methods [9] have attracted significant attention in the field of agricultural pest control due to their unique physicochemical properties, high biological activity, and environmental compatibility [10,11,12]. Previous studies have shown that AgNPs exhibit significant insecticidal activity against various insects. Their effects may not be mediated by a single target, but rather may result from the combined influence of the nanoparticles themselves, the release of silver ions (Ag^+^), and surface-capping molecules, among other factors [13,14]. The toxic mechanism of AgNPs typically involves disruption of cell membrane integrity, inhibition of key enzyme activity, and accumulation of reactive oxygen species (ROS), which in turn lead to energy metabolism imbalance, oxidative stress, and cellular damage [15,16]. Compared to traditional physical and chemical synthesis methods, plant-mediated synthesis methods mediated by plant extracts can be carried out under milder conditions while reducing environmental risks [17,18]. To date, plant-mediated AgNPs have demonstrated good control efficacy against mosquitoes, lepidopteran pests, and other groups [19,20]; however, research on their application in the control of *S. invicta* remains limited, and systematic studies on the metabolic responses they induce in *S. invicta* and the underlying basis of their potential mode of action are still lacking.

Pyrethrins are natural broad-spectrum insecticidal active compounds extracted from pyrethrum flowers (*Tanacetum cinerariifolium*) [21]. They are widely used because of their highly effective knockdown activity and low toxicity to mammals [22,23]. However, pyrethrins are prone to photodegradation and biodegradation in the environment, resulting in a short residual efficacy and limited effectiveness in field applications [24,25,26]. Pyrethrins and their derivatives are rich in diverse reactive functional groups, including ester, carbonyl, and unsaturated double-bond moieties, which endow them with the potential to participate in silver ion reduction and to form a stable capping layer on the surface of nanoparticles [27,28]. Therefore, the plant-mediated synthesis of AgNPs using pyrethrins as a reducing agent, together with an evaluation of the indoor toxic activity and environmental stability of the resulting nanoparticles against *S. invicta* workers and a preliminary exploration of the metabolic effects of Pyr-AgNPs on *S. invicta* through LC-MS-based untargeted metabolomics, may provide a useful basis for understanding the toxic effects of Pyr-AgNPs and for assessing their potential application in the sustainable management of *S. invicta*.

## 2. Results

### 2.1. Characterization of Silver Nanoparticles

**Figure 1:**
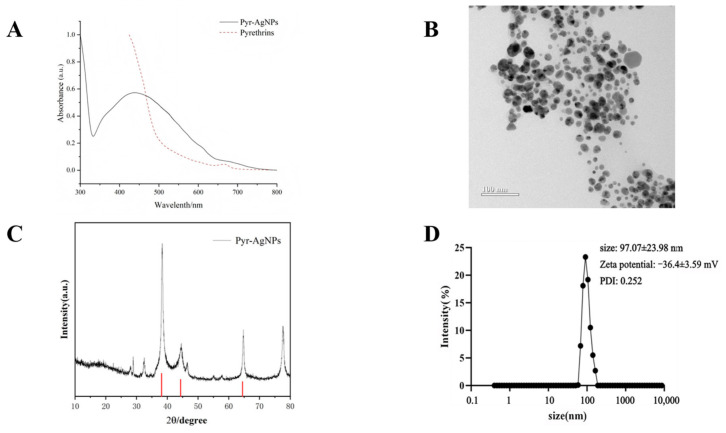
Characterization of silver nanoparticles (AgNPs): (**A**) UV–Vis absorption spectrum of biosynthesized AgNPs; (**B**) TEM image of biosynthesized AgNPs; (**C**) X-ray diffraction (XRD) pattern of biosynthesized AgNPs. The red vertical lines mark the standard diffraction peak positions of crystalline silver (JCPDS No. 87-0717). (**D**) DLS characterization of biosynthesized AgNPs.

The UV–Vis spectroscopy results (Figure 1A) showed that the pyrethrins solution exhibited no characteristic absorption in the 300–800 nm range, whereas the Pyr-AgNPs colloidal solution exhibited a typical surface plasmon resonance absorption peak at 430–460 nm, with a maximum absorption wavelength of 447.0 nm. TEM images (Figure 1B) showed that Pyr-AgNPs were primarily spherical or nearly spherical and were dispersed relatively uniformly; particle size statistics (*n* = 100) indicated an average particle size of 21.05 nm. The XRD pattern (Figure 1C) showed distinct diffraction peaks near 2θ = 38°, 44°, and 64°, corresponding to the (111), (200), and (220) crystal planes of the face-centered cubic structure of metallic silver [29], consistent with JCPDS No. 87-0717 [30], indicating that the product possessed good crystallinity. DLS (Figure 1D) measurements indicated that the average hydrodynamic particle size of Pyr-AgNPs was 97.07 nm; their polydispersity index (PDI) was 0.252, and the zeta potential was −36.4 mV, indicating that these nanoparticles exhibited good dispersion and colloidal stability in an aqueous dispersion system. The above results demonstrated that pyrethrins successfully served as a reducing agent to mediate the plant-mediated synthesis of AgNPs.

### 2.2. The Toxicity of Pyr-AgNPs to Solenopsis invicta Workers

**Figure 2:**
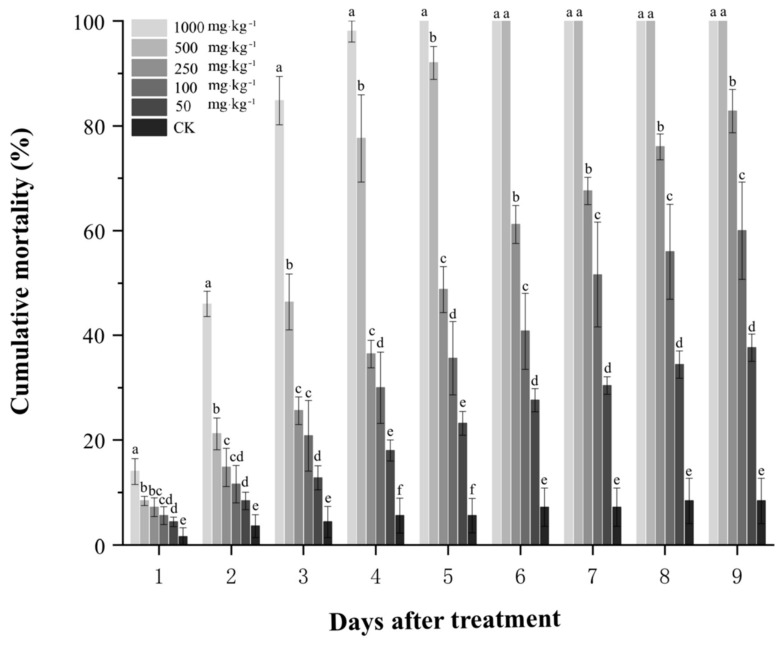
Cumulative mortality of *S. invicta* after 9 days silver of dietary administration of silver nanoparticles (AgNPs). Data are presented as the mean ± standard error (S.E.) (*n* = 5). Different lowercase letters above the bars at the same time point indicate significant differences among treatments at different concentrations, as determined by one-way analysis of variance (ANOVA) followed by the least significant difference (LSD) test (*p* < 0.05).

The mortality dynamics of *S. invicta* workers following treatment with different concentrations of Pyr-AgNPs were revealed (Figure 2). The mortality rate of *S. invicta* workers continued to rise over time following treatment with Pyr-AgNPs. The mortality rate increased most rapidly in the 1000 mg·kg^−1^ treatment group, reaching 100% by 5 d; while the 500 mg·kg^−1^ treatment group reached 100% on 6 d. The cumulative mortality rates in the 250 mg·kg^−1^ and lower concentration treatment groups increased relatively slowly and remained lower than those of the high-concentration treatment groups by the end of the experiment. The mortality rate in the CK group remained at a consistently low level, and the cumulative mortality rates of all treatment groups at each observation time point were significantly higher than those of the CK group (*p* < 0.05). These results indicated that Pyr-AgNPs exhibited high lethal activity against *S. invicta*, with clear concentration- and time-dependent effects.

The results of toxicity assays for *S. invicta* workers using Pyr-AgNP baits at different concentrations are shown (Table 1). After 9 days of treatment, the cumulative adjusted mortality rates in the 1000 and 500 mg·kg^−1^ treatment groups were both 100.00%; when the concentration was reduced to 250 mg·kg^−1^, the toxicity decreased significantly (*p* < 0.05). The cumulative adjusted mortality rates in all treatment groups were higher than those in the control group. After treatment with a 5 mmol·L^−1^ AgNO_3_ solution, the cumulative mortality rate showed no significant difference compared to the control group, indicating that AgNO_3_ is non-toxic to *S. invicta* workers. In addition, the pyrethrins bait caused a cumulative corrected mortality of 100.00% after 9 days, with an LT_50_ value of 1.625 d, indicating rapid and strong toxicity to *S. invicta* workers under the present experimental conditions. Regression analysis results showed that the 5-d LC_50_ of Pyr-AgNPs for *S. invicta* workers was 116.83 mg·kg^−1^, indicating strong toxicity to *S. invicta* workers.

### 2.3. Analysis of the Stability of Pyr-AgNPs Toxicity to Solenopsis invicta Workers

The toxicity of Pyr-AgNPs baits against *S. invicta* workers remained high during outdoor exposure and declined only after prolonged exposure (Table 2). Residual toxicity was assessed at a fixed bait concentration of 1000 mg·kg^−1^, which caused 100.00% cumulative corrected mortality in the indoor bioassay. After 24 h of outdoor exposure, the adjusted mortality caused by the pyrethrins baits decreased significantly (*p* < 0.05), and the LT_50_ increased rapidly with increasing exposure time. In contrast, Pyr-AgNPs bait still caused 100% cumulative adjusted mortality after 96 h of outdoor exposure; however, its toxicity declined significantly after 120 h of exposure (*p* < 0.05). These results indicated that Pyr-AgNPs exhibited good stability in terms of toxicity.

### 2.4. Metabolomic Analysis of Solenopsis invicta Workers Following Treatment with Silver Nanoparticles

#### 2.4.1. PCA Revealed Clear Metabolic Separation Among Different Treatments

**Figure 3:**
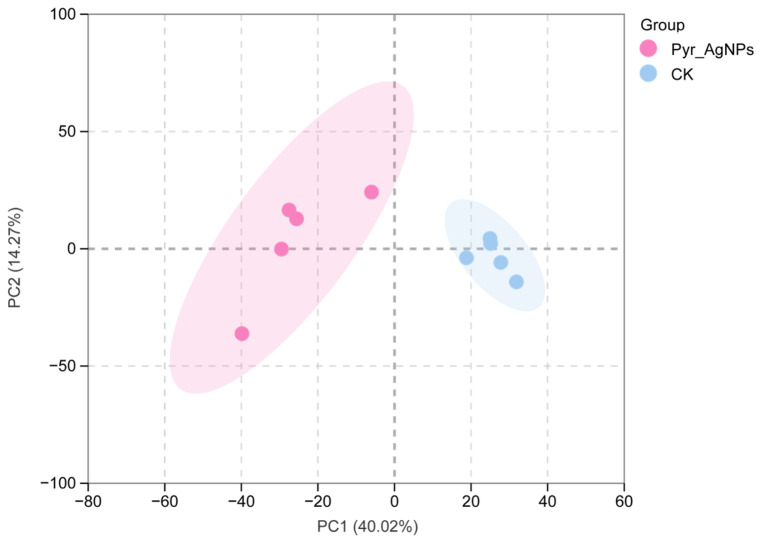
Principal component analysis (PCA) score plot of the metabolic profiles of *S. invicta* workers in the Pyr-AgNPs treatment group and the blank control (CK) group (*n* = 5). The pink dots represent the Pyr-AgNPs treatment group, and the light blue dots represent the CK group.

PCA showed that the treatment group and the blank control group (CK) were clearly separated along the PC1 axis, indicating that the metabolic profiles of *S. invicta* workers changed following treatment with Pyr-AgNPs (Figure 3). The sample points in the CK group were tightly clustered, indicating a stable metabolic state and good biological reproducibility. In contrast, the sample points in the treatment group were relatively dispersed, reflecting the instability exhibited by *S. invicta* workers in response to this stress. Thus, Pyr-AgNPs treatment had a significant impact on the metabolism of *S. invicta* workers, resulting in significant differences in metabolites compared to the CK group.

#### 2.4.2. OPLS-DA Revealed a Clear Separation Between the Pyr-AgNPs Treatment and CK Groups

**Figure 4:**
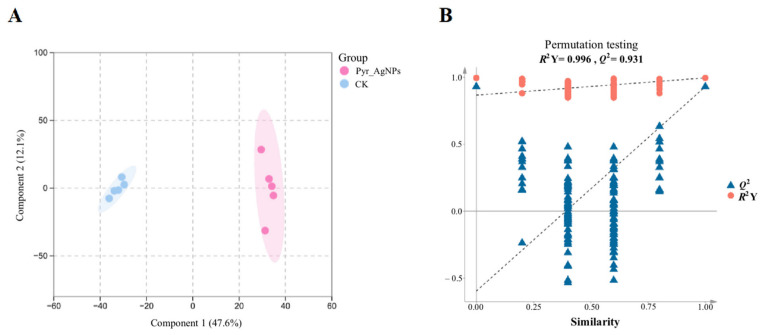
OPLS-DA score plot and model validation for the metabolic profiles of *S. invicta* workers. (**A**) OPLS-DA score plot of the Pyr-AgNPs treatment group (pink) and the blank control group (CK, light blue). (**B**) Results of the 200-fold permutation test for the OPLS-DA model. Orange circles represent *R*^2^Y and blue triangles represent *Q*^2^. The dashed lines represent the regression lines of *R*^2^Y and *Q*^2^, and their intercepts are used to evaluate the model for potential overfitting.

OPLS-DA results showed that the treatment group and the CK group were distributed in different regions on the score plot (Figure 4A), with their 95% confidence intervals not overlapping. The treatment group exhibited high separation along the PC1 principal component (explaining 47.6% of the variance). The results of the 200-fold cross-validation test (Figure 4B) showed that the model had an *R*^2^Y of 0.996 and a *Q*^2^ of 0.931, and the intercept of the *Q*^2^ regression line was less than 0. This indicated that the model had good explanatory power and did not exhibit overfitting.

#### 2.4.3. Screening for Differentially Expressed Metabolites

**Figure 5:**
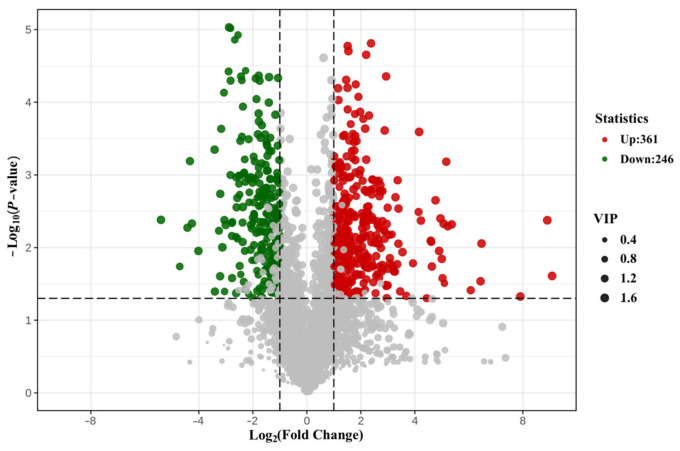
Volcano plot of differential metabolites in *S. invicta* workers treated with AgNPs. Each dot represents a single metabolite. Significantly up-regulated metabolites (361) are shown in red, and significantly down-regulated metabolites (246) are shown in green. Grey dots represent metabolites that were not significantly different between the treatment and control groups. Differentially expressed metabolites were screened based on the following thresholds: variable importance in projection (VIP) > 1, statistical significance (*p* < 0.05), and absolute fold change (|log_2_ FC| > 1). The horizontal dashed line indicates the *p*-value significance threshold, and the vertical dashed lines indicate the log_2_ FC boundaries. The size of each dot is proportional to its VIP score, reflecting its contribution to the differentiation between the two groups.

Using the screening criteria of VIP > 1, *p* < 0.05, and |log_2_ FC| > 1, and after excluding exogenous contaminants, a volcano plot was generated (Figure 5), identifying a total of 607 differentially expressed metabolites, of which 361 were upregulated and 246 were downregulated. The results indicated that the metabolic profile of *S. invicta* workers underwent significant changes following treatment with Pyr-AgNPs, with the number of upregulated metabolites exceeding that of downregulated metabolites.

#### 2.4.4. Hierarchical Cluster Clearly Distinguished the Pyr-AgNPs Treatment Group from the CK Group

**Figure 6:**
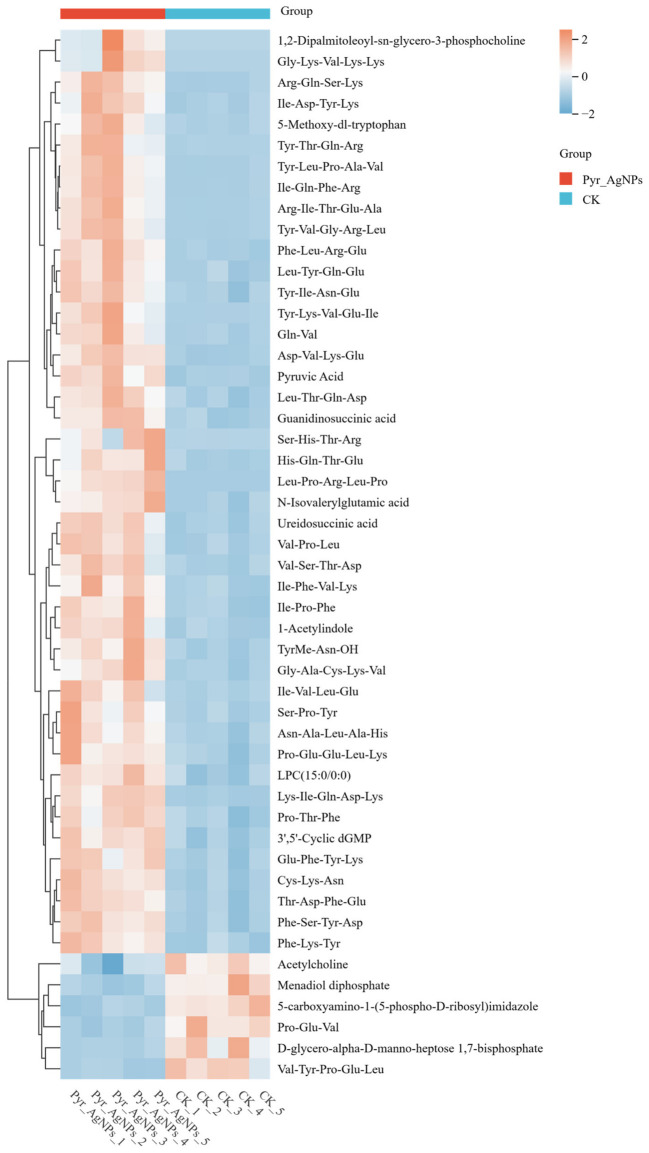
Cluster heatmap of differential metabolites in *S. invicta* workers treated with silver nanoparticles. CK represents the blank control group, and Pyr-AgNPs represents the silver nanoparticle treatment group. The numbers 1–5 on the *x*-axis indicate five biological replicates for each group.

The top 50 differentially expressed metabolites in the Pyr-AgNPs treatment group were selected in descending order based on their VIP values and subjected to hierarchical clustering analysis (Figure 6). Overall, the treatment group was clearly distinguishable from the control group, and the clustering of biological replicates within the group was relatively consistent, indicating that the metabolic profiles of *S. invicta* workers underwent significant changes following treatment with Pyr-AgNPs. In the Pyr-AgNPs group, pyruvic acid and lysophosphatidylcholine [LPC (15:0/0:0)] were upregulated, and levels of various short-chain peptide metabolites also showed an upward trend; menadiol diphosphate was downregulated. These differential metabolites were mainly involved in metabolic processes related to energy metabolism, lipid metabolism, and peptide metabolism.

#### 2.4.5. Differential Metabolites Were Mainly Enriched in Energy, Cofactor, and Carbohydrate Metabolism Pathways and ABC Transporters

**Figure 7:**
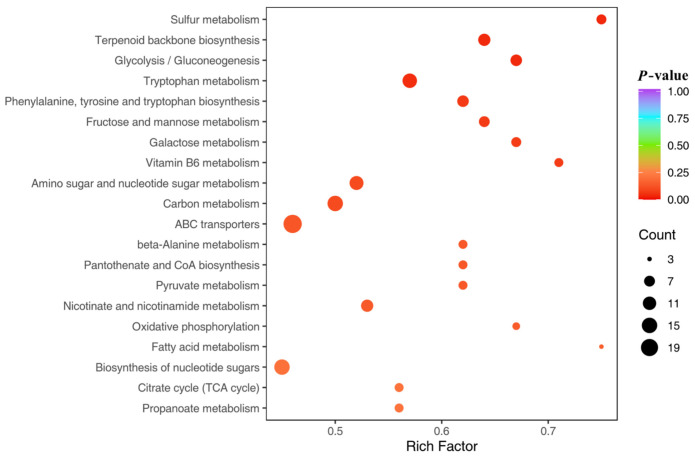
Differential metabolite enrichment pathway map of *S. invicta* workers treated with nano-silver particles.

KEGG enrichment analysis revealed that the differentially expressed metabolites in the Pyr-AgNPs treatment group were primarily enriched in the Citrate Cycle/TCA Cycle, oxidative phosphorylation, glycolysis/gluconeogenesis, sulfur metabolism, pyruvate metabolism, Pantothenate and CoA biosynthesis, Nicotinate and nicotinamide metabolism, Fructose and mannose metabolism, Galactose metabolism, Carbon metabolism, and ABC transporters (Figure 7). Thus, the differential metabolic pathways in *S. invicta* workers following treatment with Pyr-AgNPs primarily involved energy metabolism, cofactor metabolism, carbohydrate metabolism, and substance transport processes.

## 3. Discussion

In this study, pyrethrins were used as a reducing agent to synthesize Pyr-AgNPs. A characteristic surface plasmon resonance (SPR) absorption peak was observed at 447.0 nm, which falls within the typical SPR range reported for silver nanoparticles [31]. TEM analysis showed that the prepared particles were predominantly spherical or nearly spherical, with an average particle size of 21.05 nm. XRD analysis further indicated a face-centered cubic crystal structure, suggesting that the prepared particles exhibited good crystallinity. DLS and zeta potential results indicated good dispersion and stability. Collectively, these results support the successful formation of pyrethrins-mediated silver nanoparticles and indicate that pyrethrins-associated bio-derived molecules can participate in the reduction and stabilization of Ag^+^ during nanoparticle synthesis [32]. Nevertheless, because the loading content, encapsulation efficiency, and surface chemical composition of pyrethrins were not determined, the precise role of pyrethrins in nanoparticle formation remains unresolved.

The indoor toxicity bioassay showed that Pyr-AgNPs exhibited strong toxicity toward *S. invicta* workers, with an LC_50_ of 116.83 mg·kg^−1^ after 5 d of treatment. This high toxicity may be attributed to the small particle size and high surface reactivity of the silver nanoparticles. Previous studies have shown that AgNPs may involve particle-specific actions, Ag^+^ release, and interactions with surface-associated molecules, and these effects have been associated with oxidative injury, membrane perturbation, and metabolic disturbance [33,34]. In the present study, Pyr-AgNPs showed strong insecticidal activity, but because bare AgNPs were not included as controls, the relative contributions of the silver nanoparticle core and pyrethrins could not be separated, and any synergistic interaction remains speculative [35]. Field exposure results showed that 1000 mg·kg^−1^ Pyr-AgNPs bait retained high toxicity after 96 h of natural exposure, whereas pyrethrins bait at the same concentration declined significantly after 24 h, indicating that Pyr-AgNPs possessed superior environmental stability and residual efficacy. One possible explanation is that the silver nanoparticle core and surface-associated components may provide partial protection of active ingredients and alter their release behavior, thereby slowing degradation or loss of activity [36,37,38]. This interpretation is broadly consistent with previous studies showing that nanodelivery systems can improve the stability and bioavailability of plant-derived pesticides [13,39]. However, because the pyrethrins-to-silver ratio, Ag^+^ release kinetics, and long-term stability in the bait matrix were not measured, this interpretation remains provisional.

From an application perspective, the non-target safety of Pyr-AgNPs also warrants careful evaluation. Although pyrethrins are plant-derived insecticides and generally degrade relatively rapidly, they can still be highly toxic to pollinators and aquatic organisms [40]. Moreover, the improved stability and residual efficacy of Pyr-AgNPs observed in the present study, while beneficial for *S. invicta* control, may also prolong environmental exposure and increase the likelihood of interactions with non-target organisms, as has been noted for nanopesticides more broadly [41]. In addition, AgNP-based formulations may introduce further ecotoxicological concerns because silver nanoparticles can enter aquatic and terrestrial environments and may exert adverse effects on non-target biota [42]. Therefore, the present results support the efficacy of Pyr-AgNPs but do not allow conclusions regarding their ecological safety.

Untargeted metabolomics showed that the endogenous metabolic profile of *S. invicta* workers changed markedly after Pyr-AgNPs treatment. PCA and OPLS-DA analyses revealed clear separation between the treatment groups and the control group, indicating that Pyr-AgNPs induced substantial metabolic perturbation. Differential metabolite analysis, hierarchical clustering, and KEGG enrichment further suggested that the altered metabolic pathways mainly involved glycolysis/gluconeogenesis, pyruvate metabolism, the tricarboxylic acid cycle, oxidative phosphorylation, sulfur metabolism, cofactor metabolism, carbohydrate metabolism, and ABC transporters. Taken together, these results suggest that the effects of Pyr-AgNPs were associated with alterations in energy metabolism, redox homeostasis, and membrane lipid metabolism. Some of these changes may reflect direct toxic effects of Pyr-AgNPs, whereas others may represent physiological responses of the insects aimed at compensation, repair, or detoxification. This interpretation is consistent with previous reports showing that AgNPs can induce metabolic disturbances in insects through multiple physiological pathways [43]. However, these interpretations are based on metabolite variation and pathway enrichment analysis and therefore represent pathway-level inferences rather than direct physiological confirmation.

Importantly, the metabolomics design of the present study imposes a major limitation on mechanistic interpretation. Because pyrethrins-only and bare-AgNPs-only groups were not included in parallel, the present dataset cannot determine whether the observed metabolic perturbations were mainly caused by the silver nanoparticle core, by pyrethrins, or by the combination of both. Nor can it establish whether loading pyrethrins onto AgNPs changes the metabolic response qualitatively or quantitatively relative to either component alone. Therefore, the current metabolomics results support only the conclusion that Pyr-AgNPs as a formulation induced marked metabolic disturbance in *S. invicta* workers.

At the level of energy metabolism, pyruvate was significantly upregulated in the treatment group. As a central metabolic node linking glycolysis to mitochondrial aerobic oxidation, this change may reflect altered carbon flux allocation and cellular energy conversion [44]. The enrichment of glycolysis/gluconeogenesis, pyruvate metabolism, the citric acid cycle, and oxidative phosphorylation further suggests that Pyr-AgNPs may disrupt mitochondrial-related energy metabolism [45,46]. Previous studies have found that AgNPs can damage mitochondrial structure [47], disrupt the electron transport chain, and induce reactive oxygen species accumulation, thereby limiting ATP synthesis and disturbing cellular energy homeostasis [48,49]. At the same time, it should be noted that remodeling of central carbon metabolism can also occur as a secondary response, as intoxicated insects may reallocate energy resources to sustain survival, repair, and detoxification. Therefore, the present results suggest mitochondrial and energy-metabolic involvement, but they do not distinguish direct mitochondrial injury from downstream metabolic compensation. The present results also suggest that Pyr-AgNPs may be associated with oxidative stress and membrane lipid disturbance. LPC (15:0/0:0) was significantly upregulated, and its accumulation may indicate altered membrane lipid homeostasis because LPC is linked to phospholipid turnover, membrane remodeling, and reduced membrane stability [50,51]. In this context, LPC accumulation could be interpreted in two, not mutually exclusive, ways: it may reflect membrane damage caused by the toxic action of Pyr-AgNPs, and it may also represent a secondary remodeling response of the insect to restore membrane integrity after injury. Moreover, AgNPs and released Ag^+^ can trigger excessive ROS production and lipid peroxidation, thereby compromising membrane integrity [52]. The enrichment of sulfur metabolism and the abnormal level of menadiol diphosphate further suggest disruption of redox homeostasis [53,54]. Together, these findings are consistent with the possibility that Pyr-AgNPs induce redox imbalance accompanied by membrane lipid disturbance. However, direct evidence for oxidative damage and membrane injury was not obtained in the present study.

Several limitations of the present study should be acknowledged. The roles of pyrethrins in nanoparticle formation were not fully resolved, the respective contributions of pyrethrins and the silver nanoparticle core to the observed insecticidal activity remain unclear, and the ecological safety of Pyr-AgNPs toward non-target organisms was not evaluated. In addition, the metabolomics results provide pathway-level evidence, but direct physiological and biochemical validation is still needed. Accordingly, future studies should combine quantitative loading analysis and detailed surface characterization, comparative bioassays with bare AgNPs and formal synergy analysis, non-target risk assessment, and targeted biochemical and ultrastructural approaches to better clarify the formation mechanism, mode of action, environmental behavior, and safety profile of Pyr-AgNPs.

## 4. Materials and Methods

### 4.1. Test Insects

Colonies of the red imported fire ant (*Solenopsis invicta* Buren) were collected from a sugarcane field at Fujian Agriculture and Forestry University (26°08′04.7″ N, 119°23′07.1″ E). The colonies used in this study were specifically collected from the field for this experiment and were not derived from laboratory-maintained colonies. The collected colonies included queens, reproductive males, and workers. The colonies were transferred to plastic rearing boxes (40 cm × 30 cm × 10 cm), and the inner walls were coated with talcum powder to prevent escape. The colonies were reared indoors at 26 ± 1 °C and 60–80% relative humidity and were provided with 10% (*w*/*v*) sugar solution during the experimental period.

Before the experiments, the colonies were reared in the laboratory for at least 7 days. *S. invicta* workers used in the bioassays were selected from these laboratory-maintained colonies. Individuals with generally similar body size and morphology were selected, thoroughly mixed, and randomly assigned to replicate units. Because the exact age of individual workers could not be determined, age uniformity was not strictly controlled.

### 4.2. Chemicals and Reagents

The reagents used in this study included pyrethrins biopesticide (active ingredient: 1.5%; Beijing Qingyuanbao Biotechnology Co., Ltd., Beijing, China), methanol (chromatographic grade; Merck KGaA, Darmstadt, Germany), silver nitrate (99.8%; Sinopharm Chemical Reagent Co., Ltd., Shanghai, China), acetone (analytical grade; Sinopharm Chemical Reagent Co., Ltd., Shanghai, China), ammonium acetate (LC-MS grade; CNW Technologies, Shanghai Amply Experimental Technology Co., Ltd., Shanghai, China), acetonitrile (LC-MS grade; Shanghai Xingke High-Purity Solvents Co., Ltd., Shanghai, China), and formic acid (LC-MS grade; Aladdin Reagent (Shanghai, China) Co., Ltd., Shanghai, China).

### 4.3. Biosynthesis and Characterization of Silver Nanoparticles

#### 4.3.1. Synthesis Method

5 mL of pyrethrins biopesticide suspension was added to 95 mL of 5 mmol·L^−1^ silver nitrate (AgNO_3_) solution. The pH of the mixture was monitored using a pH meter (Shanghai INESA Scientific Instrument Co., Ltd., Shanghai, China) and adjusted to 6.0 by adding dilute HCl or NaOH dropwise as required. The mixture was then reacted in a water bath at 80 °C for 80 min, with shaking every 20 min. After cooling to room temperature, the product was centrifuged (8000 rpm, 15 min). The resulting precipitate was washed with distilled water and centrifuged (8000 rpm, 15 min) three times. Finally, Pyr-AgNPs powder was obtained via freeze-drying.

#### 4.3.2. Characterization of Physical and Chemical Properties

Characterization of Pyr-AgNPs was performed using ultraviolet-visible (UV–Vis) spectroscopy, transmission electron microscopy (TEM; FEI Tecnai G2 F30, FEI, Hillsboro, OR, USA), X-ray diffraction (XRD; D8 ADVANCE, Bruker, Karlsruhe, Germany), and dynamic light scattering/zeta potential analysis (DLS; Zetasizer Nano ZS90, Malvern Panalytical, Malvern, UK). For UV–Vis measurements, 1 mg of Pyr-AgNPs was weighed and dispersed in 2 mL of distilled water and thoroughly mixed. Distilled water was used as the blank, and scanning was performed on a UV–Vis spectrophotometer (UV2600, Shanghai Shunyu Hengping Scientific Instrument Co., Ltd., Shanghai, China) that had been preheated for 20 min and baseline corrected. Each sample was tested three times, and the data were processed using Origin 2022 software. For TEM observation, Pyr-AgNPs were dispersed in anhydrous ethanol and ultrasonically dispersed; an appropriate amount of the suspension was then dropped onto a carbon-supported copper mesh. After standing for 5 min, excess liquid was removed with filter paper, and the sample was allowed to air-dry at room temperature before observation and image acquisition. For XRD analysis, 0.3 g of Pyr-AgNPs powder was ground and loaded into the sample holder. Analysis was performed at room temperature using Cu Kα radiation (λ = 1.5406 Å); the test conditions were 40 kV and 40 mA, with a scan range of 2θ = 10–90° and a scan rate of 4°/min, and the diffraction patterns were recorded. For DLS and zeta potential measurements, 20 mg of Pyr-AgNPs was weighed, dispersed in 2 mL of distilled water, and thoroughly mixed before being injected into the sample cell. A Zetasizer Nano ZS90, preheated for 30 min, was used to measure the hydrodynamic particle size and zeta potential at 25 °C. Each sample was measured three times, with each measurement consisting of three measurement cycles.

### 4.4. Indoor Toxicity Assay of Silver Nanoparticles on Solenopsis invicta Workers

Ma et al. [35] described the bait preparation as follows: 50 g of corn flour, 30 g of wheat flour, 6 g of fish meal, and 4 g of peanut oil were weighed, and a small amount of distilled water was added to form granules with diameter of 1.2–1.5 mm. Pyr-AgNPs were dispersed in analytical-grade acetone and added dropwise to the bait mixture. After completely evaporated of the acetone, five bait formulations containing Pyr-AgNPs at 50, 100, 250, 500, and 1000 mg·kg^−1^ were obtained. Pyrethrins were diluted 1:1000 according to the manufacturer’s instructions and incorporated into the bait mixture in the same manner to prepare the pyrethrins bait. In addition, a 5 mmol·L^−1^ AgNO_3_ solution was added dropwise to the bait to produce AgNO_3_ bait containing 1000 mg·kg^−1^ of AgNO_3_. This treatment was included as an independent reference control and was not incorporated into statistical comparisons across the AgNP concentration gradient, as its concentration unit differed from that used for the AgNP treatments. The control bait was prepared by adding an equal volume of analytical-grade acetone without Pyr-AgNPs.

Deng et al. [55] employed the bait method as the test method. For each replicate, 5 g of bait was placed in the center of a food tray (16 cm × 11 cm × 6 cm) with inner walls coated with talcum powder, together with a cotton ball (approximately 2 cm^3^) to maintain humidity. Approximately 50 *S. invicta* workers were transferred into each tray. Each Pyr-AgNPs concentration constituted a separate treatment, with five replicates per treatment. No formal randomization procedure was applied; however, the workers were thoroughly mixed before grouping and allocated to replicate units in a non-selective manner to minimize allocation bias. The control group was treated in the same manner. After treatment, all trays were maintained in a climate chamber at 25 ± 1 °C and 80% ± 5% relative humidity. Mortality was assessed daily at regular intervals. Workers that remain completely motionless after gentle stimulation with tweezers were considered dead. Mortality was record for each group, and observations were continued for up to 9 days or until all *S. invicta* workers in a treatment group had died. The calculation formulas are as follows:

Cumulative mortality (%) = Number of dead insects/Total number of test insects × 100(1)

Cumulative corrected mortality (%) = (Mortality in treatment group − Mortality in control group)/(1 − Mortality in control group) × 100(2)

### 4.5. Assessment of the Stability of the Toxicity of Silver Nanoparticles

Bait containing 1000 mg·kg^−1^ Pyr-AgNPs was exposed outdoors for 24, 48, 72, 96, or 120 h under natural ambient conditions. During the exposure period, the temperature ranged from 28 to 35 °C and the relative humidity range from 70% to 90%. Outdoor exposure was conducted on rain-free days under natural daylight (sunny or cloudy conditions). Daylight intensity was not quantitatively measured. After exposure, the toxicity of the bait to *S. invicta* workers was determined. A control group was treated with pyrethrins bait subjected to the same outdoor exposure conditions. The bioassay procedure was the same as described in Section 4.4.

### 4.6. Assessment of the Effects of Silver Nanoparticles on the Metabolism of Solenopsis invicta Workers

#### 4.6.1. Sample Preparation and Collection

*S. invicta* workers were reared in a climate-controlled chamber (26 ± 1 °C, 80 ± 5% RH) for at least one week. The treatment group was fed bait containing 250 mg·kg^−1^ Pyr-AgNPs for 3 days, whereas the control group (CK) received bait without AgNPs for the same duration. The concentration of 250 mg·kg^−1^ was selected based on the toxicity assay because it represented an intermediate toxic level that was sufficient to induce metabolic responses without causing excessive mortality, thereby allowing adequate numbers of surviving workers for sample collection. The 3-day treatment period was selected to provide sufficient time for metabolic responses to occur while maintaining adequate numbers of live *S. invicta* workers for sample collection. The experiment consisted of one treatment group and one control group, each with five biological replicates. After the treatment period, 50 mg of live *S. invicta* workers from each replicate were placed in 2 mL sterile cryovials, rapidly frozen with liquid nitrogen, and stored at −80 °C for subsequent metabolic analysis.

#### 4.6.2. Metabolomic Sample Preparation and Liquid Chromatography-Mass Spectrometry Analysis

Samples were retrieved from a −80 °C freezer, thawed on ice until partially thawed, then chopped into small pieces. Twenty milligrams were weighed and mixed thoroughly. The mixture was homogenized in a ball mill for 20 s, centrifuged at 4 °C and 3000 rpm for 30 s, and then 400 μL of 70% methanol-water internal standard extraction solution was added. The mixture was shaken at 1500 rpm for 5 min, let it stand on ice for 15 min, centrifuge at 4 °C and 12,000 rpm for 4 min, and take 200 μL of the supernatant for injection into the LC-MS system for analysis [56].

An ultra-high-performance liquid chromatography-quadrupole time-of-flight tandem mass spectrometry system (UPLC-QTOF-MS/MS; LC-30A, Shimadzu, Kyoto, Japan; TripleTOF 6600+, SCIEX, Framingham, MA, USA) for untargeted metabolomics analysis. Chromatographic separation was performed using a Waters ACQUITY Premier HSS T3 column (1.8 μm, 2.1 mm × 100 mm; Waters, Milford, MA, USA) at a column temperature of 40 °C, with the autosampler maintained at 4 °C, a flow rate of 0.4 mL/min, and an injection volume of 4 μL. In positive ion mode, mobile phase A was 0.1% formic acid in water and mobile phase B was acetonitrile; in negative ion mode, mobile phase A was 5 mmol/L ammonium acetate in water (pH 8.0) and mobile phase B was acetonitrile. The gradient elution program was as follows: 0–1.0 min, 2% B; 1.0–9.0 min, 2–98% B; 9.0–11.0 min, 98% B; 11.0–11.1 min, 98–2% B; 11.1–14.0 min, 2% B equilibrium. Mass spectrometry was performed using an electrospray ionization (ESI) source, with data collected in both positive and negative ion modes; the spray voltages were 5500 V and −4500 V, respectively, with an ion source temperature of 550 °C, GS1 at 50 psi, GS2 at 60 psi, CUR at 35 psi, and DP at 80 V and −80 V, respectively. Data acquisition was performed in information-dependent acquisition (IDA) mode.

### 4.7. Statistical Analysis of Data

Raw data were organized using Excel 2024 and statistically analyzed with IBM SPSS Statistics v. 26.0. Probit regression analysis was performed to estimate the median lethal time (LT_50_) and 5-d median lethal concentration (LC_50_), and to calculate the toxicity regression equations and their 95% confidence intervals (SPSS Statistics v. 26.0). Cumulative mortality and cumulative corrected mortality were analyzed by one-way analysis of variance (ANOVA), followed by LSD multiple comparisons. Percentage data were arcsine square-root transformed before statistical analysis. All data were expressed as the mean ± standard deviation (mean ± SD), and differences were considered statistically significant at *p* < 0.05. Plots were generated using Origin 2022 (OriginLab Corporation, Northampton, MA, USA). Raw mass spectrometry data were converted into mzML format using ProteoWizard (v. 3.0.22172) and processed using the XCMS package (v. 3.12.0) for extraction, alignment, and retention time correction, followed by missing value filtering and imputation and support vector regression (SVR)-based peak area correction; metabolites were then identified and annotated using in-house, public, and predictive databases, and differential metabolites were screened by principal component analysis (PCA) [57], orthogonal partial least squares discriminant analysis (OPLS-DA) [58], and *t*-tests, while volcano plot [59,60], clustering heatmap analysis [61], and KEGG enrichment analysis [62], and hierarchical clustering heatmaps were generated in R v.4.6.1.

## 5. Conclusions

In this study, pyrethrins-mediated silver nanoparticles (Pyr-AgNPs) prepared via a plant-mediated synthesis route exhibited typical characteristics of silver nanoparticles, with good dispersibility and stability. Bait bioassays showed that Pyr-AgNPs exhibited strong toxicity against *S. invicta* workers. In addition, Pyr-AgNPs showed good environmental stability and residual efficacy, as bait containing 1000 mg·kg^−1^ Pyr-AgNPs still caused 100% cumulative corrected mortality after 9 days following 96 h of outdoor exposure, significantly outperforming the pyrethrins treatment. LC-MS-based untargeted metabolomics further demonstrated that Pyr-AgNPs markedly altered the metabolic profile of *S. invicta* workers, with 607 differential metabolites identified and the affected pathways mainly involving glycolysis/gluconeogenesis, pyruvate metabolism, the tricarboxylic acid cycle, oxidative phosphorylation, sulfur metabolism, cofactor metabolism, carbohydrate metabolism, and ABC transporters. Taken together, these findings suggest that the toxic effects of Pyr-AgNPs may be associated with disruption of energy metabolism, redox homeostasis, and membrane lipid metabolism. Overall, this study provides experimental support for the potential application of pyrethrins-mediated silver nanoparticle formulations in the green control of *S. invicta*.

## Figures and Tables

**Table 1:** Toxicity of Pyr-AgNPs bait at different concentrations against *S. invicta* workers after 9 days.

Concentration (mg·kg^−1^)	Treatment	Time-Mortality Regression Equation	Cumulative Corrected Mortality (%)	LT_50_ (d)	Coefficient of Determination
1000	Pyr-AgNPs	y = −1.19 + 4.46x	100.00 ± 0.00 a	1.869	0.979
500	Pyr-AgNPs	y = −1.85 + 4.26x	100.00 ± 0.00 a	2.851	0.952
250	Pyr-AgNPs	y = −1.71 + 2.56x	81.14 ± 4.55 b	4.23	0.962
100	Pyr-AgNPs	y = −1.71 + 2.04x	57.46 ± 7.88 c	6.85	0.987
50	Pyr-AgNPs	y = −1.77 + 1.54x	32.89 ± 1.96 d	-	0.993
	Pyrethrins	y = −0.7 + 3.35x	100.00 ± 0.00 a	1.625	0.977
	CK			-	
	AgNO_3_			-	

Note: Data are presented as the mean ± standard error (S.E.). Different letters indicate significant differences among treatments at the same observation time (*p* < 0.05), based on one-way analysis of variance (ANOVA) followed by the least significant difference (LSD) test (*p* < 0.05). LT_50_ values were compared separately based on their 95% confidence intervals. In the LT_50_ column, “-” indicates that the corrected mortality rate of *S. invicta* workers was less than 50.0% within 9 days; thus, the LT_50_ could not be estimated. In the time-mortality regression equations, x represents log10 (time in days). Mortality in CK and AgNO_3_ groups were 8.80% and 10.67%, respectively. Corrected mortality was calculated by Abbott’s formula.

**Table 2:** Toxicity stability of Pyr-AgNPs bait (1000 mg·kg^−1^) against *S. invicta* workers after different periods of outdoor exposure.

Treatment	Exposure Time (h)	Time-Mortality Regression Equation	9-Day Cumulative Corrected Mortality (%)	LT_50_ (d)	Determination Coefficient
Pyr-AgNPs	0	y = −1.19 + 4.46x	100.00 ± 0.00 a	1.869	0.979
	24	y = −1.29 + 4.30x	100.00 ± 0.00 a	2.047	0.970
	48	y = −1.67 + 4.00x	100.00 ± 0.00 a	2.715	0.956
	72	y = −1.51 + 3.34x	100.00 ± 0.00 a	2.831	0.981
	96	y = −1.61 + 3.41x	100.00 ± 0.00 a	3.027	0.967
	120	y = −1.5 + 2.61x	84.07 ± 3.28 b	3.818	0.990
Pyrethrins	0	y = −0.7 + 3.35x	100.00 ± 0.00 a	1.625	0.977
	24	y = −0.92 + 1.76x	76.11 ± 6.52 b	3.355	0.998
	48	y = −1.36 + 1.14x	47.74 ± 1.98 c	-	0.984
	72	y = −1.23 + 0.82x	28.32 ± 5.64 d	-	0.962
	96	y = −1.32 + 0.79x	23.45 ± 5.09 d	-	0.981
	120	y = −1.2 + 0.69x	22.50 ± 3.02 d	-	0.980
CK	0			-	

Note: Data are presented as the mean ± standard error (S.E.). Different letters indicate significant differences among outdoor exposure times within the same treatment, based on one-way analysis of variance (ANOVA) followed by the least significant difference (LSD) test (*p* < 0.05). LT_50_ values were estimated from daily mortality data using time-mortality regression analysis, and comparisons among LT_50_ values were based on the overlap of their 95% confidence intervals; non-overlapping intervals were considered significantly differences. In the LT_50_ column, “-” indicates that corrected mortality of *S. invicta* workers was less than 50.0% within 9 days; thus, the LT_50_ could not be estimated. The mortality in the CK group was 9.67%, and corrected mortality was calculated by Abbott’s formula. In the regression equations, x represents log10(exposure time in days). LT_50_ values were estimated from daily mortality records over the 9-day bioassay rather than from the final 9-day cumulative mortality alone; therefore, treatments with the same final 9-day mortality may still have different LT_50_ values.

## Data Availability

The original contributions presented in this study are included in the article. Further inquiries can be directed to the corresponding author.
